# Hepatotoxicity associated with the use of teriflunomide in a patient with multiple sclerosis: A case report

**DOI:** 10.1097/MD.0000000000028246

**Published:** 2021-12-23

**Authors:** Cristiane Munaretto Ferreira, Erica Freire de Vasconcelos-Pereira, Vanessa Marcon de Oliveira, Pedro Rippel Salgado, João Américo Domingos, Maria Tereza Ferreira Duenhas Monreal, Elvira Maria Guerra-Shinohara, Vanessa Terezinha Gubert

**Affiliations:** a Pharmacy Post-Graduation Program, Faculty Pharmaceutical Sciences, Food and Nutrition, Federal University of Mato Grosso do Sul, Campo Grande – MS, Brazil; b Pharmacy School Profª Ana Maria Cervantes Baraza, Faculty Pharmaceutical Sciences, Food and Nutrition, Federal University of Mato Grosso do Sul, Campo Grande – MS, Brazil; c Faculty of Medicine, Federal University of Mato Grosso do Sul, Campo Grande – MS, Brazil.

**Keywords:** adverse drug reaction, case report, drug-induced liver injury, multiple sclerosis, pharmacovigilance

## Abstract

**Rationale:**

Teriflunomide is an inhibitor of pyrimidine synthesis available as a first-line treatment for relapsing-remitting multiple sclerosis. Drug-induced liver damage is a relevant problem in clinical practice, representing a frequent cause of treatment discontinuation. This case report describes the occurrence of liver injury, with a 33.7-fold increase in the upper limit of normality of the liver enzyme alanine aminotransferase during treatment with teriflunomide 14 mg.

**Patient concern:**

A 44-year-old woman receiving teriflunomide 14 mg for the treatment of multiple sclerosis presented symptoms suggestive of liver dysfunction 54 days after starting treatment. The patient had no history of using disease-modifying therapy, neither previous liver disease nor other comorbidities.

**Diagnostics:**

The suggested diagnosis was drug-induced liver injury, classified as hepatocellular. Other possible hepatic and autoimmune etiologies were ruled out.

**Interventions:**

Replacement of teriflunomide treatment with glatiramer acetate and follow-up of the disease.

**Outcomes:**

Signs and symptoms regressed after treatment with teriflunomide 14 mg was discontinued, with normalization of liver enzyme activity in ∼5 months. The causality assessment of the adverse drug reaction was determined by the Naranjo scaling system, resulting in probable, with a final score of 7.

**Conclusions:**

Teriflunomide-induced liver injury in patients with multiple sclerosis is a serious adverse reaction. The report of this case contributes to updating knowledge about the safety aspects of treatment with teriflunomide and planning of monitoring strategies and patient risk management.

## 1 Introduction

Multiple sclerosis (MS) is a chronic demyelinating disease of the central nervous system that presents in a progressive and disabling way. Although there is no curative therapy for this condition, the greater availability of drugs that modify the course of the disease has considerably improved the prognosis of patients with MS. This availability favors individualized and clinically appropriate treatment. However, concerns regarding the management and monitoring of the safety aspects of therapies still persist.^[1,2]^

Teriflunomide (Aubagio, Sanofi Genzyme) is an immunomodulatory drug for oral administration that has been available for the treatment of the relapsing-remitting form of MS since 2012 worldwide.^[3]^ In Brazil, it was incorporated into the Unified Health System (UHS) in 2017. This drug is part of the Clinical Protocol and Therapeutic Guidelines as a first-line therapeutic option at a dose of 14 mg/day.^[4]^ It acts by selectively and reversibly inhibiting the mitochondrial enzyme dihydroorotate dehydrogenase, thus leading to a decrease in the proliferation of B and T lymphocytes.^[3]^ As shown in controlled studies, its risk-benefit profile is favorable, with most adverse events classified as mild and self-limited.^[5,6]^ The transient increase in alanine aminotransferase (ALT) activity in up to three times the upper limit of normality was previously described. It is mainly expected in the first 6 months of treatment with this drug, with frequencies between 14.2% and 15.8%.^[7]^ The need to discontinue the use of teriflunomide for this reason is described in up to 3% of cases.^[8]^ This report describes a case of liver injury in a patient with MS receiving teriflunomide 14 mg for <2 months.

## 2 Report

A 44-year-old brown woman, born in the state of Mato Grosso do Sul, Brazil and residing in the capital city of the state, diagnosed with relapsing-remitting multiple sclerosis, presented visual loss and diplopia, in addition to weakness in the right hemibody and paresthesia on the right, with a score of 2.0 on the Kurtzke Expanded Disability Status Scale. Patient was normotensive, with a body mass index of 23.1 kg/m^2^, laboratory tests of renal and hepatic function without alterations as well as metabolic and hematological profiles. The diagnosis of relapsing–remitting multiple sclerosis (RRMS) was confirmed in July 2019 in a specialized medical service linked to the Brazilian UHS based on the McDonald Criteria revised in 2017.^[1]^ The patient had not received any previous disease-modifying therapy. Patient family history of illnesses was unknown as she was an adopted. She denied alcohol consumption, drug use and smoking. No history of previous liver disease or other comorbidities. Obstetric history of pregnancy at 19 years of age and surgical removal of the gallbladder in 2018 due to the presence of stones. Allergies to the drugs ibuprofen, metoclopramide, bromopride, scopolamine butylbromide, and tramadol had been reported. She denied the use of other medications or herbal medicines. The patient clinical details use was authorized in written informed consent and the Research Ethics Committee linked to the Federal University of Mato Grosso do Sul approved the publication of this case report.

In August 2019, the patient started treatment for MS with teriflunomide 14 mg orally once a day and vitamin D supplementation 60,000 international unit (IU) daily, with pharmacotherapeutic follow-up provided by the local Clinical Pharmacy Service. After 54 days of using the drug, the patient attended the pharmaceutical consultation reporting abdominal pain, loss of appetite, vomiting, and nausea, with worsening of the condition for a week. She denied concomitant use of other medications or herbal medicines. She was advised to seek an emergency care service for laboratory tests, returning to the pharmacy after 2 days with results of ALT 1384 U/L, aspartate aminotransferase (AST) 612 U/L, gamma-glutamyl transferase (GGT) 690 U/L, total bilirubin 1.20 mg/dL, and direct bilirubin 0.50 mg/dL. Suspecting an adverse reaction related to the recently started drug, the patient was referred for medical evaluation at the neurology service, where she was already undergoing follow-up for MS. The case was considered probable hepatotoxicity associated with the use of teriflunomide, adopting the suspension of treatment as a conduct. This adverse drug reaction was notified to the Brazilian Pharmacovigilance Service, and the causality assessment was quantified by the Naranjo scaling system,^[9]^ whose final score was 7, indicating that the drug was probably associated with the reaction.

During outpatient follow-up, in addition to monitoring liver parameters, some tests were performed to assess other possible etiologies of liver disease. Serological tests for hepatitis B and C, Epstein–Barr virus, cytomegalovirus, syphilis, and HIV infections were negative, as were anti-smooth muscle, antinuclear and hepatic-renal membrane antibodies. The patient's rheumatologic profile was normal. Thirty days after treatment interruption with teriflunomide 14 mg, there was a gradual improvement of the condition, with values for AST 146 U/L, ALT 144 U/L, GGT 380 U/L, alkaline phosphatase (AF) 159 U/L, TB 0.50 mg/dL, and DB 0.40 mg/dL. The diagnosis of drug-induced liver injury (DILI) was suggested after ruling out other possible etiologies of liver disease.

In November 2019, the patient started a new treatment for MS with 20 mg/mL glatiramer acetate (Copaxone, Teva Pharmaceutical) daily use subcutaneously. When attending the return medical appointment two months after starting therapy with 20 mg/mL glatiramer acetate, the patient still had a mild elevation of liver enzymes, with the following results for AST 78 U/L, ALT 70 U/L, GGT 114 U/L, and AF 104 U/L. Other tests, including total bilirubin and fractions, metabolic profile, hematology and renal function, were within normal limits. At that time, the pharmacological presentation of glatiramer acetate was changed to 40 mg/mL subcutaneously, three times a week, for greater patient dosage convenience. Normalization of all liver tests (AST 19 U/L; ALT 16 U/L, GGT 26 U/L, FA 71 U/L, BT 0.60, and BD 0.1 mg/dL) was observed in March 2020. The patient's clinical condition remained stable, and the last Magnetic resonance imaging of the brain performed for the evolutionary control of the disease (March 2021) did not show changes in the image pattern of the lesions.

## 3 Discussion

In this report, a case of DILI in a 44-year-old woman was described, with a 33.7-fold increase in the upper limit of normality (ULN) of the liver enzyme ALT after 54 days of treatment with teriflunomide 14 mg, which led to discontinuation of therapy. The regression of signs and symptoms after discontinuation of the drug and the probably association with the reaction indicated by the Naranjo assessment strengthened the cause-effect relationship with teriflunomide. Furthermore, no other potentially hepatotoxic drugs or supplements were started by the patient, and other possible hepatic and autoimmune etiologies were ruled out. To the best of our knowledge, only one other case of abruptly increased ALT activity has been reported. This is a 32-fold increase in the ULN of ALT in a 35-year-old woman 5 months after starting teriflunomide therapy, with severe liver damage.^[3]^ What stands out in our case report was the precocity of alteration in ALT activity, which occurred in <2 months and at values higher than the case previously described in the literature.

DILI is a relevant problem in clinical practice, representing a frequent cause of treatment discontinuation. Before establishing the diagnosis of DILI, it is important to rule out other liver disease etiologies. In this case report, it was possible to classify DILI as hepatocellular type, since it fulfilled the criteria of serum ALT activity twice above the ULN.^[10]^

The occurrence of DILI may be common to disease-modifying therapies used for MS, either in platform therapy (interferon-β or glatiramer acetate) or in newer drugs, such as alemtuzumab, teriflunomide, and fingolimod.^[11,12]^ The early removal of the drug causing liver damage is the indicated clinical management.^[10]^

Despite the paucity of real-world publications regarding the safety profile of teriflunomide, the risk of severe liver damage was previously associated with its precursor leflunomide, which has been used in the treatment of rheumatoid arthritis since 1988.^[3]^ Other commonly reported adverse drug reaction caused by teriflunomide includes gastrointestinal symptoms, hair loss, headaches, and increased blood pressure.^[5–7]^

It is noteworthy that under normal conditions, the elimination process of a drug takes 4 to 6 half-lives, since the presence of liver dysfunction or reduced serum albumin levels can contribute to prolonging the elimination process.^[13]^ In this case report, teriflunomide was discontinued, as it is considered the most likely cause of liver damage in the patient, taking ∼5 months for normalization of liver tests after discontinuation of treatment. As teriflunomide has a half-life of 19 days,^[3]^ the delay in normalizing the activity of liver enzymes may be related to their long permanence in the plasma circulation, since an accelerated elimination procedure was not adopted to remove the drug. Additionally, it is important to consider that the exact period until the normalization of liver tests cannot be accurately estimated, as laboratory monitoring of liver tests was not performed weekly.

As a treatment strategy for MS in this patient, glatiramer acetate was prescribed, and daily vitamin D supplementation continued (60,000 IU/day). Glatiramer acetate has been used in the treatment of MS since 1996, and liver changes are not common during this therapy.^[11]^ This drug acts by inducing the production of anti-inflammatory type II antigen-presenting cells and secretion of cytokines that promote a neuroprotective effect.^[12]^ Regarding the therapeutic use of vitamin D in the treatment of MS, it is suggested that supplementation can contribute to the reduction of inflammatory processes through a modulating action on the immune system. However, there is no solid scientific evidence to demonstrate the effectiveness of this monotherapy in controlling the disease.^[14]^

The identification, primarily by the Clinical Pharmacy Service, of the causal relationship between the symptoms and the start of drug use demonstrates the importance of pharmaceutical health care. Pharmacovigilance and pharmacotherapeutic follow-up actions can contribute to better clinical outcomes, quality of life and safety for patients with MS, especially in the context of new therapies and complex therapeutic regimens.^[15]^ DILI is an adverse drug reaction that can be detected and reported.

## 4 Conclusion

In conclusion, teriflunomide-induced liver injury in patients with MS is a serious adverse reaction. It is important to report this life-threatening reaction as well as monitoring liver function parameters (serum transaminases, bilirubin, and alkaline phosphatase) during treatment. The report of this case contributes to updating knowledge about the safety aspects of treatment with teriflunomide and planning of monitoring strategies and patient risk management.

## Author contributions

**Conceptualization:** Cristiane Munaretto Ferreira, Erica Freire de Vasconcelos-Pereira, Vanessa Marcon de Oliveira.

**Formal analysis:** Elvira Maria Guerra-Shinohara, Pedro Rippel Salgado.

**Investigation:** Cristiane Munaretto Ferreira, Erica Freire de Vasconcelos-Pereira, Pedro Rippel Salgado, João Américo Domingos.

**Supervision:** Vanessa Marcon de Oliveira, Elvira Maria Guerra-Shinohara, Vanessa Terezinha Gubert.

**Writing – original draft:** Cristiane Munaretto Ferreira, Erica Freire de Vasconcelos-Pereira, Vanessa Marcon de Oliveira, Elvira Maria Guerra-Shinohara.

**Writing – review & editing:** Cristiane Munaretto Ferreira, Erica Freire de Vasconcelos-Pereira, Maria Tereza Ferreira Duenhas Monreal, Vanessa Terezinha Gubert.
